# Graph states of prime-power dimension from generalized CNOT quantum circuit

**DOI:** 10.1038/srep27135

**Published:** 2016-06-07

**Authors:** Lin Chen, D. L. Zhou

**Affiliations:** 1 School of Mathematics and Systems Science, Beihang University, Beijing 100191, China; 2 International Research Institute for Multidisciplinary Science, Beihang University, Beijing 100191, China; 3 Beijing National Laboratory for Condensed Matter Physics, and Institute of Physics, Chinese Academy of Sciences, Beijing 100190, China

## Abstract

We construct multipartite graph states whose dimension is the power of a prime number. This is realized by the finite field, as well as the generalized controlled-NOT quantum circuit acting on two qudits. We propose the standard form of graph states up to local unitary transformations and particle permutations. The form greatly simplifies the classification of graph states as we illustrate up to five qudits. We also show that some graph states are multipartite maximally entangled states in the sense that any bipartition of the system produces a bipartite maximally entangled state. We further prove that 4-partite maximally entangled states exist when the dimension is an odd number at least three or a multiple of four.

Maximal entanglement is the key ingredient in quantum teleportation, computing and the violation of Bell inequality. The maximally entangled state of two qubits can be created by controlled-phase gate or controlled-not (CNOT) gate. In this sense, they have the same power to create entanglement. In fact, the two gates are related by local Hadamard gates. As we know, only one type of two-qubit unitary gates and single qubit gates are enough to build a universal quantum circuit. A natural idea is to use those gates to generate maximally entangled states in many qubit case^1^,^2^,^3^. The graph states and cluster states are generated by applying two-qubit phase gates to an initially product state^4^. Single-qubit gates are not involved in the generation. So the quantum circuit to create graph states is composed of controlled phase gates only. The graph states and continuous-variable cluster states are constructed to study one-way quantum computing^4^,^5^,^6^,^7^. They are useful for self-testing of nonlocal correlations^8^ and their entanglement can be effectively evaluated by the Schmidt measure^9^, relative entropy of entanglement and the geometric measure of entanglement^10^,^11^. Recently the graph states have been generalized to prime dimensions even in continuous variables, in terms of the encoding circuit and Hadamard matrices^12^ and quantum codes and stabilizers^13^. The cluster states can also be defined using finite groups and controlled-phase gates^14^,^15^.

The Hilbert space of prime-power dimensions has been studied for a few quantum-information problems, such as the mutually unbiased basis, the stabilizer code and the Clifford group underlying distillation^16^. In this paper we study the multi-qudit graph state when the dimension *d* = *p*^*m*^ is a power of a prime number *p*. It ensures the existence of finite field structure, and at the same time generalizes^12^. With the aid of the structure, generalized CNOT gates are defined naturally. A general *N* qudit state generated by a quantum circuit is constructed in Eq. (88). To simplify this state, we propose a standard form of multiqubit state in Eq. (89). Our first main result is Theorem 1, stating that the above two families are equivalent up to local unitary transformations and particle permutations. We also propose the dual graph state of the standard form in (94), and show that they are equivalent under local unitary transformation in Theorem 2. It further simplifies the structure of multiqudit graph states, and we classify them up to five parties.

Our main task is to find out the maximally entangled state by the quantum circuit composed of generalized CNOT gates. The task induces a preliminary problem: what states are called maximally entangled states of many-qudit system? The basic requirement is that any single qudit is entangled with the other systems. We further require that the many-qudit state is a maximally entangled state if any bipartition of systems produces a bipartite maximally entangled state^1^,^2^,^3^,^17^,^18^. We will show that some graph states are multipartite maximally entangled states. We further prove that 4-partite maximally entangled states exist when the dimension is an odd number at least three or a multiple of four. This is another main result in our paper, as stated in Theorem 3. These results imply that the maximal entanglement is universal in high dimensions. We also construct a connection between the maximal entanglement and an entropy problem recently proposed in^19^.

This paper is organized as follows. First we will introduce the generalized CNOT in the qudit case with the aid of the structure of finite field, and then a quantum circuit composed pure generalized CNOT gates is given. Next we prove that only bipartite graph states can be generalized from the quantum circuit of pure generalized CNOT gates. Third we analyze the maximal entanglement of these states. Finally, we give a summary of our results and open problems.

## Quantum Circuit of Pure Generalized CNOT Gates

In this section we construct the generalized CNOT gates by two one-qudit operations *A*(*a*_*m*_) and *D*(*a*_*m*_). They are mathematically realized by the known finite field and the commutation relations. Using the CNOT gates we construct the quantum circuit. We will introduce a standard form of *N*-qudit graph state on finite field in (89), and show that any graph state is equivalent to the standard form up to local unitary transformations and particle permutations. To obtain a simpler classification of such states we propose Theorem 2 and demonstrate it by states up to five systems respectively in the figures.

### Finite field and generalized CNOT gates

As is well known, when *d* is the power of a prime number, i.e.,


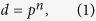


where *p* is prime, and *n* is a positive integer, there is a field *F*_*d*_. Note that the field *F*_*d*_ is unique up to isomorphism. The elements of the Field *F*_*d*_ are denoted as {*a*_*i*_, *i* ∈ {0, 1, …, *d* − 1}}, where *a*_0_ ≡ 0 and *a*_1_ ≡ 1 are the units for the sum and the product operations respectively.

We introduce a *d*-dimensional Hilbert space *H*_*d*_ with a natural orthonormal basis {|*a*_*i*_〉}. With the aid of the sum and product operations in the field, two classes of basic one-qudit operations are defined


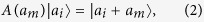



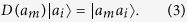


Obviously, the operation *A*(*a*_*m*_) is unitary for any *m*. If *a*_*m*_ ≠ 0, then *D*(*a*_*m*_) is also unitary.

Since *F*_*d*_ is an Abelian group under the operation +, then we have


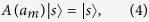


where


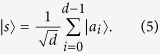


We introduce the generalized CNOT gate from qudit *m* to qudit *n* labeled by *a*_*k*_ defined by


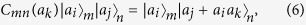


where qudit *m* is the control qudit, and qudit *n* is the target qudit.

First, we notice that


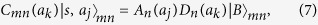


where


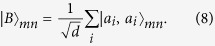


In addition, when *d* = 2 and *a*_*k*_ = 1, the gate *C*_*mn*_(1) is the CNOT gate. Therefore any *C*_*mn*_(*a*_*k*_) with *a*_*k*_ ≠ 0 is a generalized CNOT gate, which can generate the two-qudit maximal entangled state from a separable state.

### Commutation relations for related unitary transformations

Before investigating the properties of the generated states, let us first calculate the basic commutation relations for related unitary transformations widely used throughout the paper. The proof of these relations will be given in the end of this subsection. First we study the one qudit case. According to the definitions given in Eq. (2) and Eq. (3), we have


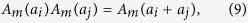



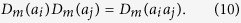


The commutation relations between *A*_*m*_ and *D*_*m*_ are


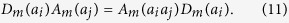


In addition, we also have


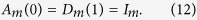


Next we study the two-qudit case. The first set of relations are


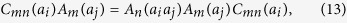



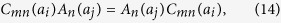



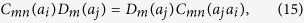



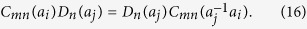


The second set of relations includes two equations. The first equation is


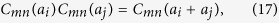


which is easy to prove but important in simplifying our graph sates. The second equation is


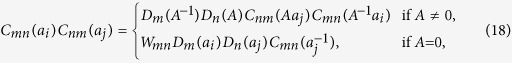


where *A* = 1 + *a*_*i*_*a*_*j*_, *W*_*mn*_ is the swap gate between the qudits *m* and *n*.

Third we study the three-qudit case. The relations for three qudits are given by


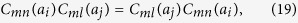



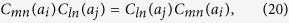



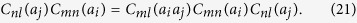


**Figure 1:**
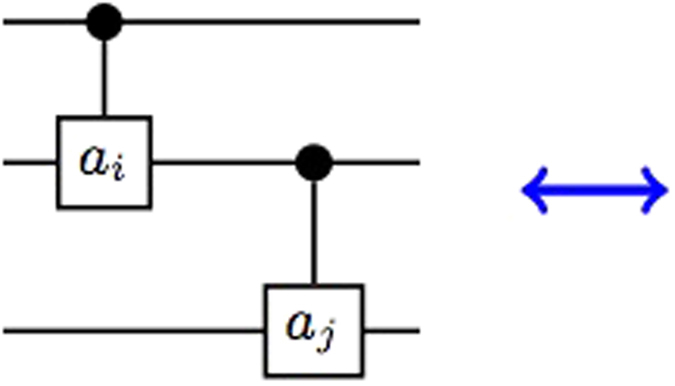
Circuit representation of Eq. (21). The gate diagram and Fig. 2 both represent the same unitary transformation.

**Figure 2:**
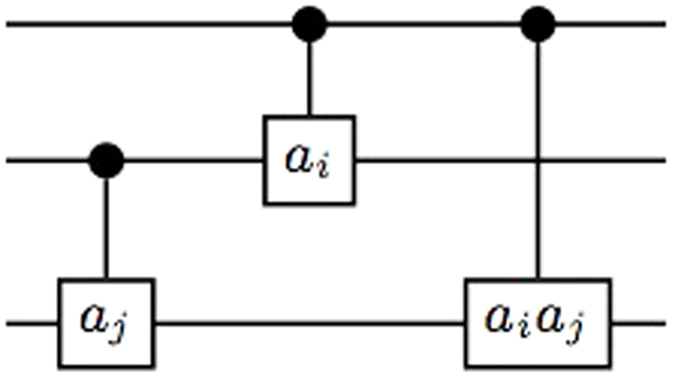
Circuit representation of Eq. (21).

Here we use two circuits to represent Eq. (21) as shown in Figs 1 and 2.

Finally we give the proof of relations in Eqs (11)–(21), respectively.

The proof of Eq. (11):

Notice that the identity operator for the *m*-th particle is


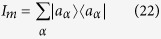



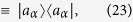


where we take the Einstein’s rule for repeated indexes. Then


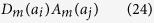



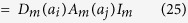



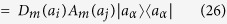



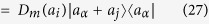



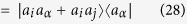



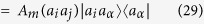



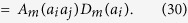


The proof of Eq. (13):


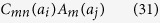



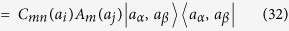



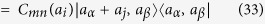



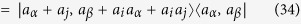



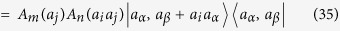



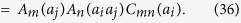


The proof of Eq. (14):


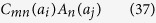



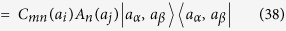



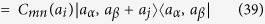



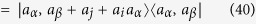



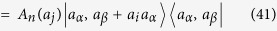



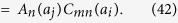


The proof of Eq. (15):


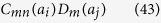



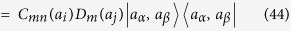



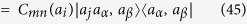



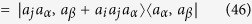



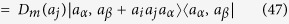



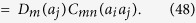


The proof Eq. (16):


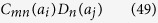



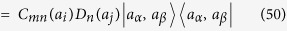



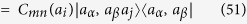



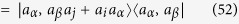



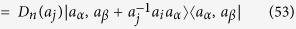



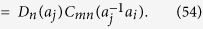


If *A* ≠ 0, then


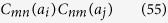



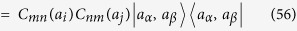



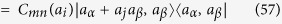



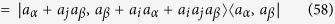



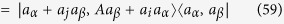



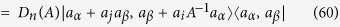



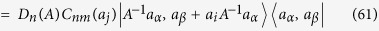



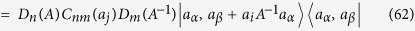



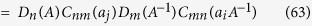



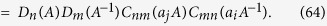


If *A* = 0, then


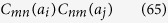



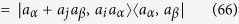



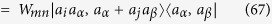



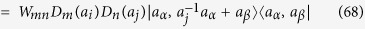



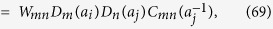


where *W*_*mn*_ is the swap gate between the *m*-th qudit and the *n*-th qudit.

The proof of Eq. (19):


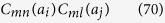



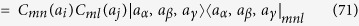



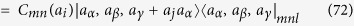



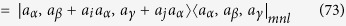



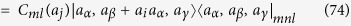



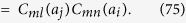


The proof of Eq. (20):


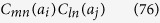



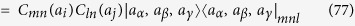



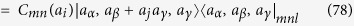



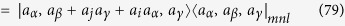



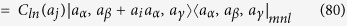



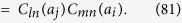


The proof of Eq. (21):


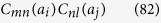



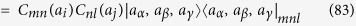



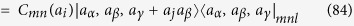



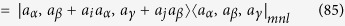



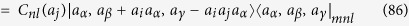



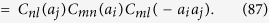


### Quantum circuit based on controlled gates

Since a controlled gate can generate a two-qudit maximally entangled state, and a two-qudit gate is enough to entangle a complex quantum circuit, a natural generalization is to apply the controlled gates to generate many-qudit maximally entangled state by a quantum circuit.

**Figure 3:**
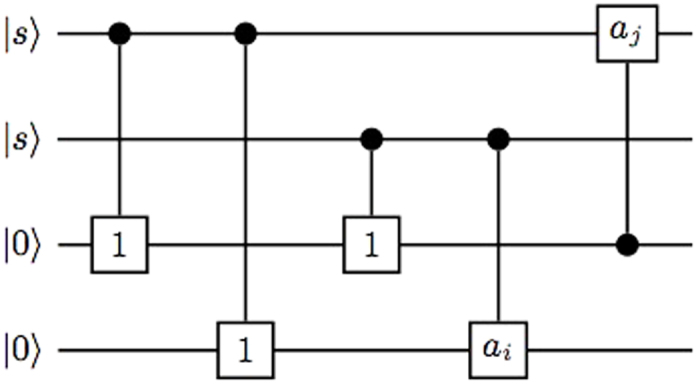
A quantum circuit to generate 4-qudit graph state.

A quantum circuit based on the controlled gates *C*_*mn*_(*a*_*k*_) is an *N*-qudit circuit with a series of controlled gates operating on, see an example as shown in Fig. 3. Up to local unitaries, a general *N* qudit (*d* = *p*^*m*^) state generated by a quantum circuit is


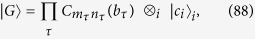


where *c*_*i*_ ∈ {*s*, 0}, *b*_*τ*_ ∈ *F*_*d*_, *τ* ∈ {1, 2,…, *M*} with *M* being the number of the controlled gates, and (*m*_*τ*_, *n*_*τ*_) ∈ {1, 2,…, *N*}.

A central problem is to investigate the possible types of entangled states through a series of the above controlled operations with some given initial states. The difficulties in simplifying the circuit lies in the facts that the number of controlled gates *M* may be very large, and these controlled gates do not commute with each other in general.

## Graph State on Finite Field

According to the initial state of an *N*-qudit circuit state in Eq. (88), we divide the *N* qudits into two sets: the set of qudits with the initial state 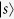 and the set of qudits with the initial state 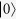, denoted as *S* and *O* respectively. The sets could be empty. Now we introduce a standard form of *N*-qudit graph state on finite field as


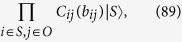


where *b*_*ij*_ ∈ *F*_*d*_, and


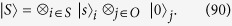


**Figure 4:**
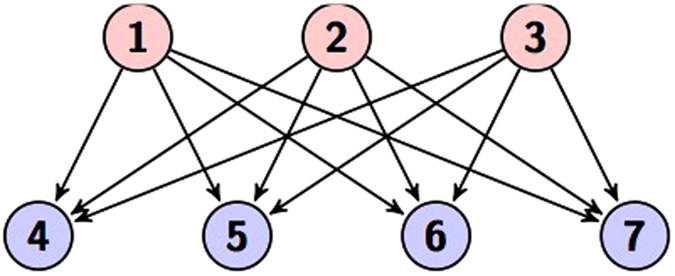
A bipartite graph state with *N* = 7 and *S* = {1, 2, 3}, and the labels {*b*_*ij*_} are omitted.

This state is called a graph state because all the controlled gates in the circuit commute, and it is can be represented as a directed bipartite graph. An example of a graph state for *N* = 7 and the set *S* = {1, 2, 3} is demonstrated in Fig. 4.

One of our central results is the following theorem:

**Theorem 1** *Any state in* Eq. (88) *is equivalent to the standard form in* Eq. (89) *up to local unitary transformations and particle permutations.*

A direct way to prove the above theorem is to show a state in the standard form under the action of any generalized CNOT gate will still be a standard one. More precisely, we only need to show


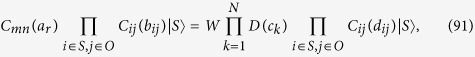


where *m*, *n* ∈ {1, 2,…, *N*}, *a*_*r*_, *b*_*ij*_, *c*_*k*_, *d*_*ij*_ ∈ *F*_*d*_, and the SWAP gate *W* represents an arbitrary particle permutations. It can be proved by directly applying the commutation relations given in the last section. As the proof is long, we give another more concise proof.

*Proof.* Let the initial state 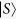 be 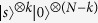 with *k* ∈ [1, *N* − 1]. Using (6) we can show that 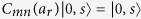 for any *a*_*r*_ ∈ **F**_*d*_. It follows from (88) that up to local unitaries any graph state can be expressed as


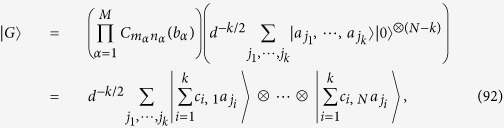


where *c*_*i*,*q*_ ∈ **F**_*d*_ is the linear combination of *b*_*α*_ by (6). The *k* × *N* matrix [*c*_*i*,*q*_] has the same rank as that of [*I*_*k*_, 0], because the former is obtained from the latter via the operation 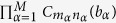. So [*c*_*i*,*q*_] has rank *k*. Up to the permutation of vertices, we may assume that the first *k* column vectors in [*c*_*i*,*q*_] are linearly independent. The last *N* − *k* column vectors in [*c*_*i*,*q*_] are the linear combinations of them. Let *b*_1,1_, ···, *b*_*k*,*N*_ ∈ **F**_*d*_ be the coefficients in the linear combination. From (92) we have


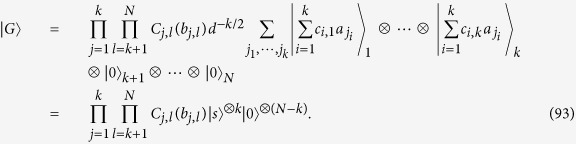


The second equality follows from the fact that 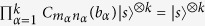 for any gate 
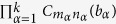
 and any *b*_*α*_ ∈ **F**_*d*_. Hence we can generate 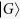 by performing the gate 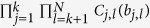 on the initial state 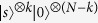. The time order of *C*_*j*,*l*_(*b*_*j*,*l*_) in the gate is random, because they commute. This completes the proof. □

The main conclusion from the above theorem is that up to local unitary transformations and particle permutations all the states generated by the controlled gate circuit are the directed bipartite graph states, and the graph contains only the edges from 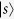 to 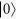, which greatly simplifies our investigations on possible types of entanglement created by the controlled gate circuit.

The dual graph state for the graph state specified by Eqs (89) and (90) is


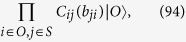


where


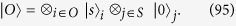


**Theorem 2.** *The two graph states given in* Eqs (89) *and* (94) *for two dual graphs are local unitary equivalent.*

*Proof.* For a finite field with *d* = *p*^*n*^ and *p* a prime, the element is represented as 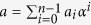, where *a*_*i*_ are *F*_*p*_ elements, represented by integers modulo *p*, i.e., *a*_*i*_ ∈ {0, 1,…, *p* − 1} and *α* is one root of the equation for an irreducible polynomial of degree *n* over the finite field with cardinality *p*. For example, when *p* = 3 and *n* = 2, the corresponding irreducible polynomial may be taken as *x*^2^ + *x* + 2, *α* is one root of *x*^2^ + *x* + 2 = 0, and any element in the field with cardinality 9 is represented as *a*_0_ + *a*_1_*α* with *a*_0_, *a*_1_ ∈ {0, 1, 2}. When *α*^*i*^ is regarded as the bases, the element in the finite field can be denoted as a vector 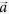. Then we introduce the discrete Fourier transformation of the states 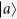 as


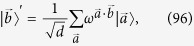


where


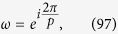



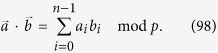


Therefore we define the Hadamard transformation as


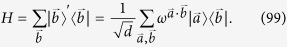


Then


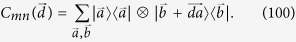


Therefore


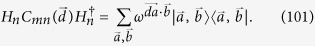


Let *α*^*j*^(*α*^*k*^) with *k* ∈ {0, 1, ···, *n* − 1} and *j* ∈ {0, 1, ···, 2 * (*n* − 1)} denote the coefficient of term *α*^*k*^ in the expression *α*^*j*^. Then we have


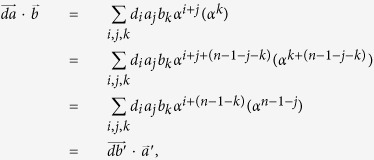


where


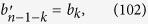



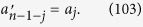


So we introduce local unitary transformation


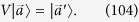


Therefore we have


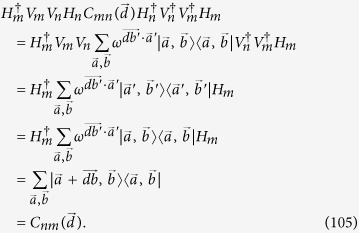


In addition,


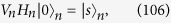



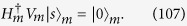


Therefore we have


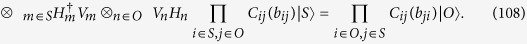


This completes our proof. □

This theorem implies that we can restrict ourselves in the cases where the cardinality of *S* is less than or equal to the cardinality of *O*, i.e. [*N*/2].

**Figure 5:**
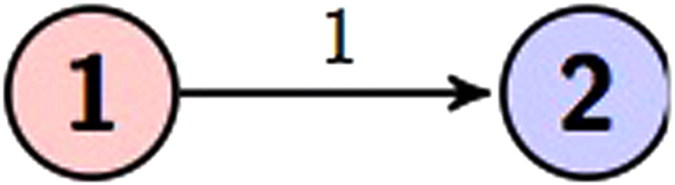
Two qudit graph.

Now let us apply the above theorems to study the possible types of entanglement generated by the controlled gates for *N* = 3, 4, 5 with the help of Eqs (15) and (16). There is only one type of two qudit graph state, which is the qudit Bell state in Fig. 5. Note that if we replace 1 by a finite field element *a*_*k*_, then we can convert the resulting state into the state by the element 1 using local unitary operations *D*(*a*_*m*_). Mathematically, Theorem 1 says that we need to investigate the state 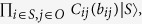 where *b*_*ij*_ ∈ *F*_*d*_ and 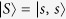, 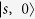 or 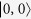. It is easy to see that the state is a product state unless 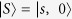. In this case, the state becomes the qudit Bell state 
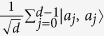
.

**Figure 6:**
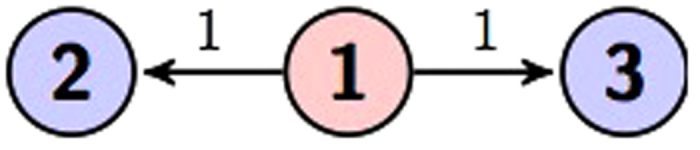
Three qudit graph.

**Figure 7:**
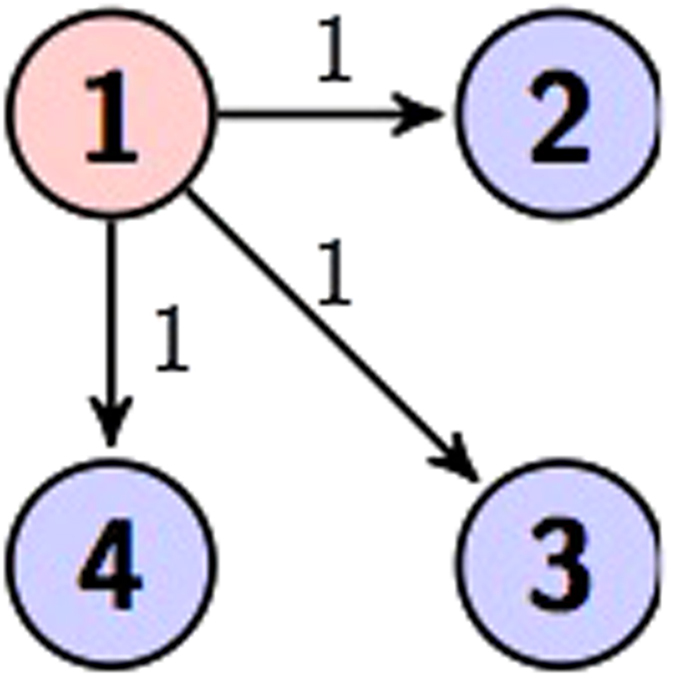
Four qudit graph states.

**Figure 8:**
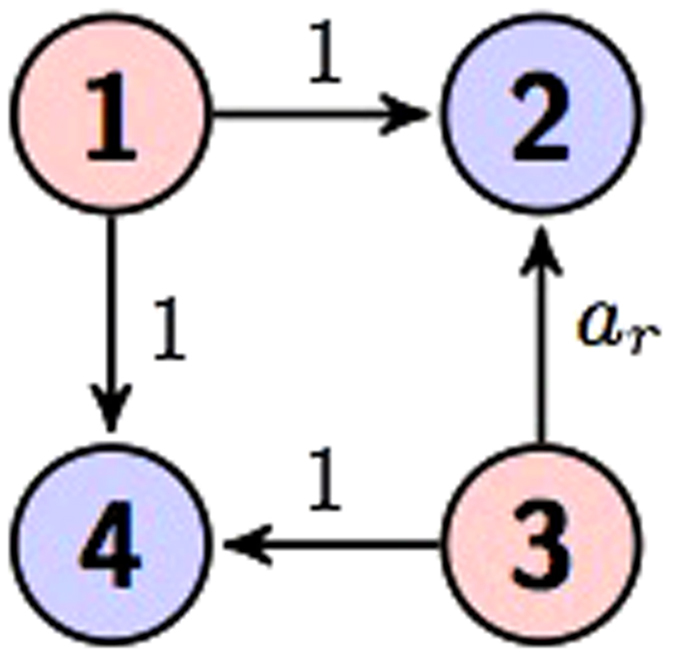
Four qudit graph states.

Similar arguments show that there is also one type of three qudit graph state, which is a generalized GHZ state in Fig. 6:

There are two types of four qudit graph states. One is four qudit GHZ state in Fig. 7. The other type in Fig. 8 has a more fruitful configuration, which will be studied in next section. As proved in Theorem 1, any graph state, say the generalized cluster state illustrated by a line connecting all vertices is locally equivalent to one of the types.

**Figure 9:**
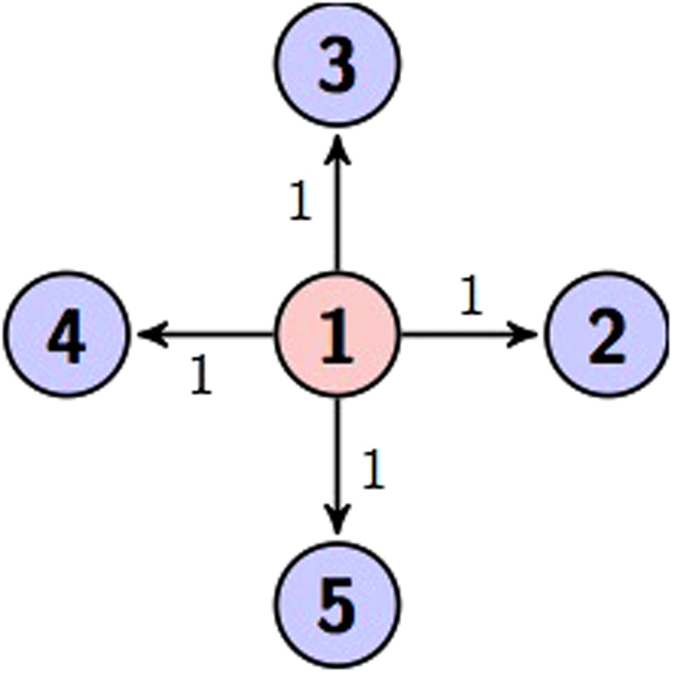
Five qudit graph states.

**Figure 10:**
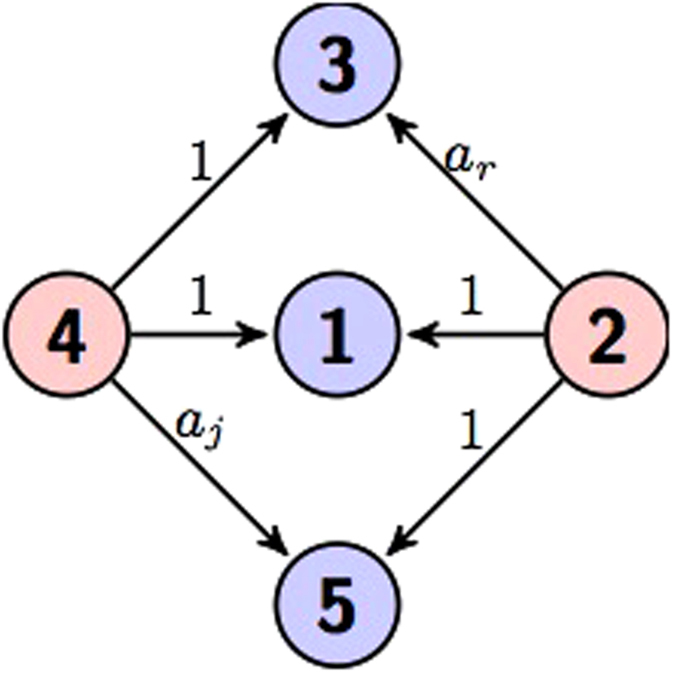
Five qudit graph states.

There are also two types of five qudit graph states in Figs 9 and 10.

## Entanglement Properties of Qudit Graph States

In this section we study the maximal entanglement of graph states defined in previous sections. The state in Fig. 8 can be written as


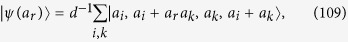


where *a*_*r*_ ∈ *F*_*d*_, the dimension *d* = *p*^*n*^ with a prime *p* and positive integer *n*. We have

**Lemma 1.** 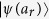 *is a maximally entangled state when a*_*r*_ ∈ *F*_*d*_\{*a*_0_, *a*_1_}.

*Proof.* Since *F*_*d*_ is a field and *a*_*r*_ ∈ *F*_*d*_\{*a*_0_, *a*_1_}, we have *F*_*d*_ = *a*_*r*_*F*_*d*_ = *a*_*i*_ + *F*_*d*_ for any *a*_*i*_ ∈ *F*_*d*_. So 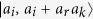 is an o. n. basis in **C**^*d*^ ⊗ **C**^*d*^. One can similarly verify that all three bipartite reduced density operators of 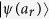 respectively w. r. t. the bipartitions 12:34, 13:24 and 14:23 are maximally mixed states. It implies that the bipartition between one particle and other three particles is a bipartite Bell state. So 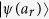 is a maximally entangled state. □

If *n* = 1 then *d* is a prime number. This case has been studied in^12^ and is a special case of the lemma. The case *d* = 2 is excluded in the lemma, and it coincides with the known result that 4-qubit maximally entangled state does not exist^2^. we demonstrate them by a simple example. We set *a*_*r*_ = 2, *a*_*j*_ = *j* and *d* = 4 in (109), and obtain


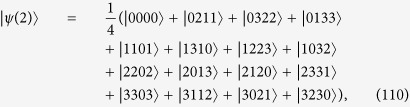


by using the computation rule in Table 1. On the other hand, Lemma 1 does not hold when *d* is replaced by any integer which is not a prime power.

Next we give an example of maximal entanglement beyond the primer-power dimension. The state


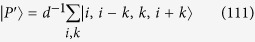


appeared in^12^, in which *d* was considered as a prime number. We point out that the state can be defined for any integer *d*. One can straightforwardly show that 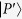 is a maximally entangled state for any odd *d* ≥ 3, and is not a maximally entangled state for any even *d* > 1. The two families of states 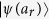 and 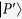 show that 4-partite maximally entangled states are universal in high dimensional spaces. Indeed we have

**Theorem 3.** *The maximally entangled 4-partite pure state exists when the dimension d is an odd number at least three, or a multiple of four.*

*Proof.* The state 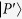 validates the assertion when *d* is an odd number at least three. So the first assertion holds. It remains to prove the second assertion when *d* is a multiple of four. We may assume 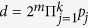 where *m* ≥ 2, *k* ≥ 0, and *p*_*j*_ ≥ 3 are prime numbers. The first assertion implies that the maximally entangled state with every system of dimension *p*_*j*_ exists. Let the state be 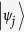 on the system *A*_*j*_*B*_*j*_*C*_*j*_*D*_*j*_ such that 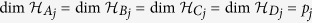. Lemma 1 implies that the maximally entangled state with every system of dimension 2^*m*^ exists. Let the state be 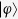 on the system *A*_0_*B*_0_*C*_0_*D*_0_ such that 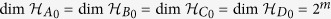. We combine the corresponding above systems to obtain a new 4-partite system *ABCD*, i.e.,


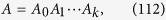



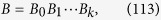



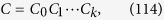



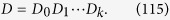


Now we construct a new 4-partite pure state 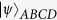 via the tensor product of corresponding states as follows


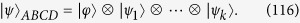


Since 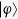 and the 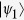
’s are all maximally entangled states, 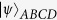 is the maximally entangled state of system dimension *d*. Since *d* is a multiple of four, the second assertion holds.□

The above proof indeed shows an analytical way of constructing the 4-partite maximally entangled states in designated dimensions. In spite of the above results, we do not have any example of 4-partite maximally entangled state with dimension equal to the multiple of two and any positive odd number. We conjecture they might not exist. This is true when the odd number is one^2^. So the first challenge is to construct a 4-partite maximally entangled state with dimension 6. It easily reminds us of the construction of mutually unbiased basis of dimension 6, which is a long-standing problem in quantum physics.

Finally as a more independent interest, we construct the connection between maximal entanglement and the entropy problem recently proposed in^19^. The problem asks to construct (or exlcude the existence of) a tripartite quantum state *ρ*_*ABC*_ such that rank*ρ*_*AB*_ > rank*ρ*_*AC*_ ⋅ rank*ρ*_*BC*_. The problem turns out to be hard and constructing the connection might be helpful to finding out its solution.

**Lemma 2.** *Let ρ*_*ABC*_ *be a tripartite state whose bipartite reduced density matrices are all maximally mixed states* 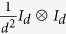
*. Then*

- *ρ*_*ABC*_ exists and rank*ρ*_*ABC*_ ≥ *d*.
- The maximally entangled 4-partite pure state exists if and only if there is a *ρ*_*ABC*_ such that rank*ρ*_*ABC*_ = *d*.

*Proof.* (i) A trivial example is 
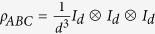
. Let 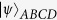 be the purification of *ρ*_*ABC*_. Then rank*ρ*_*AB*_ = rank*ρ*_*CD*_ = *d*^2^ ≤ rank*ρ*_*C*_ rank*ρ*_*D*_. Since rank*ρ*_*C*_ = *d*, we have rank*ρ*_*ABC*_ = rank*ρ*_*D*_ ≥ *d*.

(ii) We prove the “if” part. Suppose there is a tripartite state *ρ*_*ABC*_ of rank *d*, whose bipartite reduced density matrices are all maximally mixed states 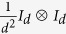. Let 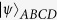 be the purification of *ρ*_*ABC*_. So 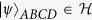 is maximally entangled. The “only if” part can be similarly proved. This completes the proof.□

## Conclusions

We have constructed multipartite graph states with prime-power dimension using the generalized CNOT quantum circuit. We have proven that the graphs states are equivalent to a simple and operational standard form up to local unitary transformations and particle permutations. We also showed that some graph states are multipartite maximally entangled states, and that 4-partite maximally entangled states exist when the dimension is an odd number at least three or a multiple of four. The next question is to study graph states defined by generalized controlled-phase gates. Another problem is to quantify the entanglement of these graphs states in terms of multipartite entanglement measures, such as the geometric measure of entanglement and relative entropy of entanglement. Constructing the potential link between maximal entanglement and the mutually unbiased basis for dimension six may be a long-term goal of receiving more attention.

## Additional Information

**How to cite this article**: Chen, L. and Zhou, D. L. Graph states of prime-power dimension from generalized CNOT quantum circuit. *Sci. Rep.* **6**, 27135; doi: 10.1038/srep27135 (2016).

## Figures and Tables

**Table 1:** The two tables respectively account for the addition and multiplication operations for *F*_4_.

+	0	1	2	3
0	0	1	2	3
1	1	0	3	2
2	2	3	0	1
3	3	2	1	0

×	0	1	2	3
0	0	0	0	0
1	0	1	2	3
2	0	2	3	1
3	0	3	1	2

The proof of Lemma 1 also holds when *F*_*d*_ is replaced by any finite domain, because it coincides with the finite field.
